# A three-dimensional actively spreading bone repair material based on cell spheroids can facilitate the preservation of tooth extraction sockets

**DOI:** 10.3389/fbioe.2023.1161192

**Published:** 2023-02-27

**Authors:** Xinwei Guo, Huimin Zheng, Yusi Guo, Boon Chin Heng, Yue Yang, Weitong Yao, Shengjie Jiang

**Affiliations:** ^1^ Beijing Laboratory of Biomedical Materials, Department of Geriatric Dentistry, Peking University School and Hospital of Stomatology, Beijing, China; ^2^ Department of Prosthodontics, The First Clinical Division, Peking University School and Hospital of Stomatology, Beijing, China; ^3^ Hubei Jiangxia Laboratory, Wuhan, China; ^4^ Guangdong Provincial Key Laboratory of Stomatology, Hospital of Stomatology, Sun Yat-Sen University, Guangzhou, Guangdong, China

**Keywords:** actively spreading material, site preservation, cell spheroid, polyether F127 diacrylate, mesenchymal stem cell

## Abstract

**Introduction:** Achieving a successful reconstruction of alveolar bone morphology still remains a challenge because of the irregularity and complex microenvironment of tooth sockets. Biological materials including hydroxyapatite and collagen, are used for alveolar ridge preservation. However, the healing effect is often unsatisfactory.

**Methods:** Inspired by superwetting biomimetic materials, we constructed a 3D actively-spreading bone repair material. It consisted of photocurable polyether F127 diacrylate hydrogel loaded with mixed spheroids of mesenchymal stem cells (MSCs) and vascular endothelial cells (ECs).

**Results:** Biologically, cells in the spheroids were able to spread and migrate outwards, and possessed both osteogenic and angiogenic potential. Meanwhile, ECs also enhanced osteogenic differentiation of MSCs. Mechanically, the excellent physical properties of F127DA hydrogel ensured that it was able to be injected directly into the tooth socket and stabilized after light curing. *In vivo* experiments showed that MSC-EC-F127DA system promoted bone repair and preserved the shape of alveolar ridge within a short time duration.

**Discussion:** In conclusion, the novel photocurable injectable MSC-EC-F127DA hydrogel system was able to achieve three-dimensional tissue infiltration, and exhibited much therapeutic potential for complex oral bone defects in the future.

## 1 Introduction

Tooth extraction is a common dental surgical procedure to deal with teeth that have lost restoration value and normal physiological functions. Missing teeth may seriously impair masticatory function and patients’ esthetic appearance, so they need to be repaired as soon as possible. However, after tooth extraction, osteoclasts are usually activated due to trauma and inflammatory granulation tissue within the tooth socket, resulting in continuous bone resorption (Jung et al., 2018). Studies have shown that alveolar bone is resorbed rapidly in both the horizontal and vertical directions within 3–6 months after tooth extraction, and that the resorption rate slows down after 6 months. Unfortunately, the whole process is irreversible (Tomlin et al., 2014). Statistics have shown that 50% of the buccolingual alveolar bone were resorbed during the first year after tooth extraction (Araújo and January 2011). If appropriate measures are not taken, rapid bone resorption can compromise prosthodontic rehabilitation. Alveolar ridge preservation (ARP) is a surgical procedure to slow down alveolar bone resorption by implanting bone increment materials into the socket, and covering it with barrier membrane materials immediately after tooth extraction (G. Avila-Ortiz et al., 2020; Majzoub et al., 2019). The most commonly used implant materials are hydroxyapatite, bio-ceramic and collagen (Kim et al., 2016; Avila-Ortiz et al., 2019). However, these materials only offer structural support in the tooth socket, and exert only very limited pro-osteogenic effects because of their inadequate biocompatibility. Moreover, implants might be treated as foreign objects by the body and trigger specific immune responses *in situ*, resulting in immune exclusion effect and even bone resorption. In recent years, stem cell therapy has been proposed to effectively reduce immune exclusion effect. Studies have shown that stem cells preferentially migrate to the defect area that displays the increased expression of inflammatory chemokines (Liesveld, 2020). Furthermore, they have the potential to differentiate into multiple lineages, such as the osteogenic and angiogenic lineages. Hence, after tooth extraction, stem cells are able to migrate to the tooth socket and play a key role in bone repair (Meng et al., 2022). Exogenous mesenchymal stem cell transplantation can speed up the repair process, but requires a large number of stem cells so that prior *ex vivo* cell expansion is necessary. However, current 2D adherent cultures often lead to rapid cell senescence after successive passage, and impair their multipotent differentiation potential (Turinetto et al., 2016), which ultimately results in reduced proliferation and osteogenic capacity after transplantation. Moreover, because of gravity, the transplanted stem cells often converge at the lowest position and cannot make full contact with the bone defect in a three-dimensional (3D) manner. In order to overcome these problems, inspired by previous studies on biomimetic superwetting materials (Jiang et al., 2021; Yang et al., 2022), we fabricated a 3D actively-spreading bone repair material. The photocurable polyether F127 diacrylate hydrogel loaded with mixed spheroids of mesenchymal stem cells (MSCs) and vascular endothelial cells (ECs) was injected into the alveolar bone defect to accelerate bone regeneration.

Biomimetic superwetting materials take inspiration from the natural structures of living organisms, therefore they have strong hydrophobicity and high biocompatibility *in vivo* (Li et al., 2021). The most imitated structure is the lotus leaf, which surface is abundant in micron-level protrusions, making itself highly hydrophobic and self-cleanable. Water can form beads on the lotus leaves, easily roll off afterwards and carry away dust (Farzam et al., 2022). Cell membranes are also hydrophobic, because it is mainly composed of a phospholipid bilayer. Due to its hydrophobic effect, it can isolate intracellular fluid from extracellular fluid, ensuring the relative stability of the cellular internal environment. Studies have shown that the hydrophobicity of cell membranes facilitates cell adhesion and infiltration. The more hydrophobic the cells are, the stronger the infiltration and adhesion will be (Rosenberg, 1991). Cell spheroids are growth forms of large numbers of cells aggregating into a 3D spherical structure. The cell spheroid itself can be used as a biologically-active infiltrating material, because the hydrophobic area of the cell membrane is expanded, allowing better infiltration within the tooth extraction socket filled with blood and saliva. Furthermore, previous studies have shown that cell spheroids have the active spreading ability, as cells inside the spheroid can migrate in all directions by budding (Yin et al., 2022). Therefore, implanting biomaterials containing cell spheroids into the complex microenvironment of tooth sockets can ensure a higher degree of cell contact with the bone wall (Maritan et al., 2017). Cell spheroid also helps preserve the “stemness” of stem cells. Compared with cells in 2D culture, MSCs cultured spherically in 3D always maintain good multipotent differentiation and proliferative potentials (Yan et al., 2014). Studies have also shown that 2D co-culture of endothelial progenitor cells with MSCs enhance the osteogenic biomarker expression of MSCs (Peng et al., 2019). On this basis, we hypothesize that 3D cultures of ECs with MSCs to form a mixed ECs-MSCs spheroid can enhance the osteogenic potential of MSCs, facilitate early vascular reconstruction in the defect area, and promote socket healing.

Biocompatible scaffolds are also required for the implantation of cell spheroids into the socket. Here, we selected polyether F127 diacrylate (F127DA), an acrylacylated polyethylene glycol—polypropylene glycol—polyethylene glycol triblock copolymer, as the 3D scaffold for spheroid implantation. F127DA has demonstrated a good biosafety record and excellent mechanical properties (Ren et al., 2019). Additionally, F127DA hydrogel is temperature-sensitive before light curing, forming a gelatinous shape at higher temperatures, and returning to the liquid state at lower temperatures. The internal temperature of the mouth is always maintained at about 37°C, and the fluidity of F127DA is reduced at this temperature, impeding it from flowing out of the socket after injection. With photo-initiator added into the system, it can be quickly cross-linked into hydrogels under ultraviolet or visible light of a certain wavelength, which can be easily operated in the dental clinic. In all, we constructed a 3D actively-spreading material incorporated with EC-MSC mixed spheroids, which demonstrated promising clinical application potential and is expected to be a favorable biomaterial for the treatment of complex oral bone defects in the future.

## 2 Materials and methods

### 2.1 Preparation and harvest of spheroids

ECs and MSCs were first cultured in T75 culture flasks. When the cell confluence reached 80%, ECs and MSCs were dissociated with trypsin. Then the cell suspension was centrifuged at a speed of 1,200 rpm. The supernatant was discarded, and cells were resuspended with fresh medium. AggreWell™400 24-well plate (STEMCELL Technologies, Canada) was prepared in an ultra-clean cabinet. The Anti-Adhesion Rinsing Solution (STEMCELL Technologies, Canada) was then added to the plate with aliquots of 500 µL per well. Then the plate was centrifuged at 1,300 g for 5 min. Microscopy observations were used to assess whether the bubbles at the bottom of the plate disappeared. If not, the plate would be re-centrifuged. Next, the Anti-Adhesion Rinsing Solution was aspirated, and the plate was then rinsed with pre-heated medium. ECs and MSCs was counted to 1.2–2.4 × 10^6^ cells per well. Cell suspensions with a volume of 2 mL were added to each well. The suspension was gently dispersed by pipetting to ensure that the cells were evenly distributed in the microwell. Then the plate was centrifuged at 100 g for 3 min, and was incubated at 37°C, 5% CO_2_ and 95% humidity for 24 or more hours. Finally, whether any spheroid was formed in the micro-well, was evaluated by observation under a microscope.

When harvesting spheroids, the supernatant was carefully aspirated from each well. 2 mL of fresh medium was added, and pipetted repeatedly to make sure that spheroids were suspended from the bottom of microwells. Medium containing spheroids was collected into a centrifuge tube. The centrifuge tube was then placed on the test-tube stand for more than half an hour, waiting for the spheroids to settle down naturally. It should be noted that the cell spheroids cannot be centrifuged, or their shape may be damaged. Then the supernatant was discarded. The remaining sediments were the target spheroids.

### 2.2 Observations of spheroid spreading behavior

The harvested MSC spheroids and MSC-EC mixed spheroids were cultured in 48-well plates. The budding of spheroids was observed and photographed every 2 h. The image processing software ImageJ FIJI was used to calculate the number and length of the budding, so as to calculate the spreading area of the spheroids at different time points.

### 2.3 RT-qPCR

The spheroids were lysed by 1 mL TRIzol (Thermo Fisher, The United States), pipetted several times and left to stand on ice for 10 min. Then 200 μL of chloroform was added into the centrifuge tube. The mixture was then centrifuged at a speed of 12,000 g at 4°C for 10 min. The supernatant at the top layer was carefully transferred to another centrifuge tube, followed by the addition of 500 μL isopropanol into the supernatant. After being rotated up and down for 10 min, the tube was centrifuged at a speed of 12,000 g at 4°C for 15 min. Then the supernatant was discarded. The RNA was washed and precipitated twice with 75% ethanol, centrifuged at the speed of 7,500 g for 5 min each time. The target RNA was then dried for 10 min at room temperature until the color turned completely transparent.

20 µL of RNase free water was then added into the centrifuge tube to dissolve RNA. A nanodrop (Thermo Fisher, The United States.) was used to measure the RNA concentration. RNA was then reverse transcribed into cDNA by a reverse transcription kit (Takara, Japan) and PCR thermal cycler (Takara, Japan). Transparent 96 well plates (Thermo Fisher, The United States.) and a real-time fluorescent quantitative PCR system (ABI, The United States.) were used for qPCR detection. The qPCR mixture with a total volume of 10 µL per well includes 5 µ L FastStart universal SYBR Green Master Mix (Roche, Germany), 3 µ L RNase free water, 1 µ L template cDNA, and 1 µ L primer. The experimental results were analyzed by relative quantitative methods. Primer sequences utilized for qRT-PCR are shown in Table 1.

**TABLE 1:** Primer sequences utilized for qRT-PCR.

GAPDH	TCT​CTG​CTC​CTC​CCT​GTT​C	ACA​CCG​ACC​TTC​ACC​ATC​T
OPG	GGT​AAT​GAC​ACG​ATC​ACT​CC	TGACACGATCACTCC
CD31	CAG​CCA​TTA​CGA​CTC​CCA​GA	GAG​CCT​TCC​GTT​CTC​TTG​GT

### 2.4 Alkaline phosphatase qualitative staining

Spheroids cultured for 3 days were harvested and cultured on 24 well plate. After 24 h, 1 mL neutral formaldehyde was added into each well to fix cells for 10 min. Then the plate was washed twice with deionized water. An alkaline phosphatase (ALP) qualitative kit (Beyotime, China) was used to stain the spheroids.

### 2.5 Alizarin red staining

Spheroids cultured for 10 days were harvested and cultured on 24 well plates. After 24 h, 1 mL of neutral formaldehyde was added into each well to fix cells for 10 min. Then the plate was washed twice with deionized water. The spheroids were then stained with 500 μL of alizarin red solution incubated on a shaker at room temperature for 15 min, and finally rinsed by deionized water again.

### 2.6 Immunofluorescence

Spheroids were seeded in a 24-well plate with glass bottom for laser confocal microscopy. The sample was rinsed with PBS and fixed with neutral formaldehyde solution for 10 min 0.5% (w/v) Triton X-100 (Sigma, The United States) was used to permeabilize the cells for 10 min. Then 3% (w/v) bovine serum albumin (BSA, Solarbio, China) was used for blocking at 4°C for 20 min. The antibodies were added to the sample and incubated overnight. Antibodies used include: Anti-CD34 antibody (ab198395, abcam, United Kingdom.), and Anti-BMP2 antibody (ab276041, abcam, United Kingdom). The cell nuclei were stained with 4′, 6-diamidino-2-phenylindole (DAPI; Sigma, The United States). The cell spheroids were observed under laser confocal microscopy and photographic images were captured.

### 2.7 Preparation of F127DA hydrogel

PBS was used to dissolve the photo-initiator. The solution was heated by water bath at 40°C–50°C for 15 min. Then the photo-initiator solution was added to the F127DA powder. The mixture was stored at 2°C–8°C for 30 min, and vibrated several times during the period. It is noted that the concentration of F127DA solution is 3%–30% (w/v). The final solution was then stored in the dark after complete dissolution. When curing, a light source with a wavelength of 405 nm was used to irradiate the F127DA solution containing the photo-initiator for 30 s.

### 2.8 Hydrogel loading with spheroids

Millex Syringe Filters (Millipore, The United States) was used to filter the F127DA solution. Spheroids were collected and resuspended within the F127DA solution. Then the spheroid suspension was added to 24 well plates. Each well was irradiated by a light source with a wavelength of 405 nm for 30 s. An appropriate volume of medium was added to each well after the light curing. Finally, the plate was incubated at 37°C.

### 2.9 Hydrogel degradation

The cell strainer was weighed with an analytical balance, and the weight was recorded as M0. The F127DA hydrogel after light curing was placed into the cell strainer and immersed into the PBS solution at 37°C. The cell strainer containing hydrogel was taken out of the PBS every 24 h, dried with filter paper to absorb water on its surface, and the overall weight was weighed as Mt. The weight measured at the first 24 h is M1. The calculation formula of degradation rate (Dt) at each time point is: Dt=(Mt-M0)/(M1-M0).

### 2.10 Rheological measurements

The rheometer (Andonpa MCR302, China) was set to shock mode, with a constant frequency of 1 Hz, the strain variation range of 0.01%–100%, and the test temperature of 25°C. The fixture was a parallel plate. Before testing, the uncured liquid hydrogel was added to the plate with a hollow round mold. Then the liquid was light cured. Next, the mold was removed.

### 2.11 Compression strength test

The compression strength was measured with the universal mechanical testing machine (SHIMADZU, Japan). The tested hydrogel was molded into a cylinder with a height of 4 mm and a diameter of 10 mm. The compression rate is 5 mm/min. Then the stress-strain curve was calculated according to the displacement and the primary load measurement was automatically recorded by a computer.

### 2.12 Animal experiment

The animal experiment was approved by the Biomedical Ethics Committee of Peking University (approval number: PUIRB-LA2022688). Specific pathogen-free male Wistar rats were divided into a blank control group (Ctrl group), F127DA hydrogel group (Gel group), hydrogel loaded with MSC spheroid group (MSC group), and hydrogel loaded with MSC-EC mixed spheroids group (Mix group), according to the random number table method. The maxillary first molars were extracted intact after intraperitoneal injection of pentobarbital at a concentration of 10 mg/mL and local injection of 2% (w/v) lidocaine plus 0.1% (w/v) epinephrine hydrochloride. Spheroids and hydrogel were injected into the extraction sockets of the corresponding groups. The extraction sockets were irradiated with a light source at 405 nm for 30 s. After 3 weeks, the rats were culled by over-anesthesia, and the maxillae were explanted and scanned by Micro CT. We chose this time point because new bone gradually began to form after 3 weeks, which was conducive to comparing the differences between groups. Then the maxillae were decalcified with a 10% (w/v) EDTA solution for 2 weeks, and then sequentially dehydrated, waxed, embedded, sectioned. Hematoxylin-eosin (HE) staining, Masson staining and immunohistochemical staining of CD31 (ab182981, abcam, United Kingdom) and COL1 (ab270993, abcam, United Kingdom) were performed afterwards.

## 3 Results

**FIGURE 1:**
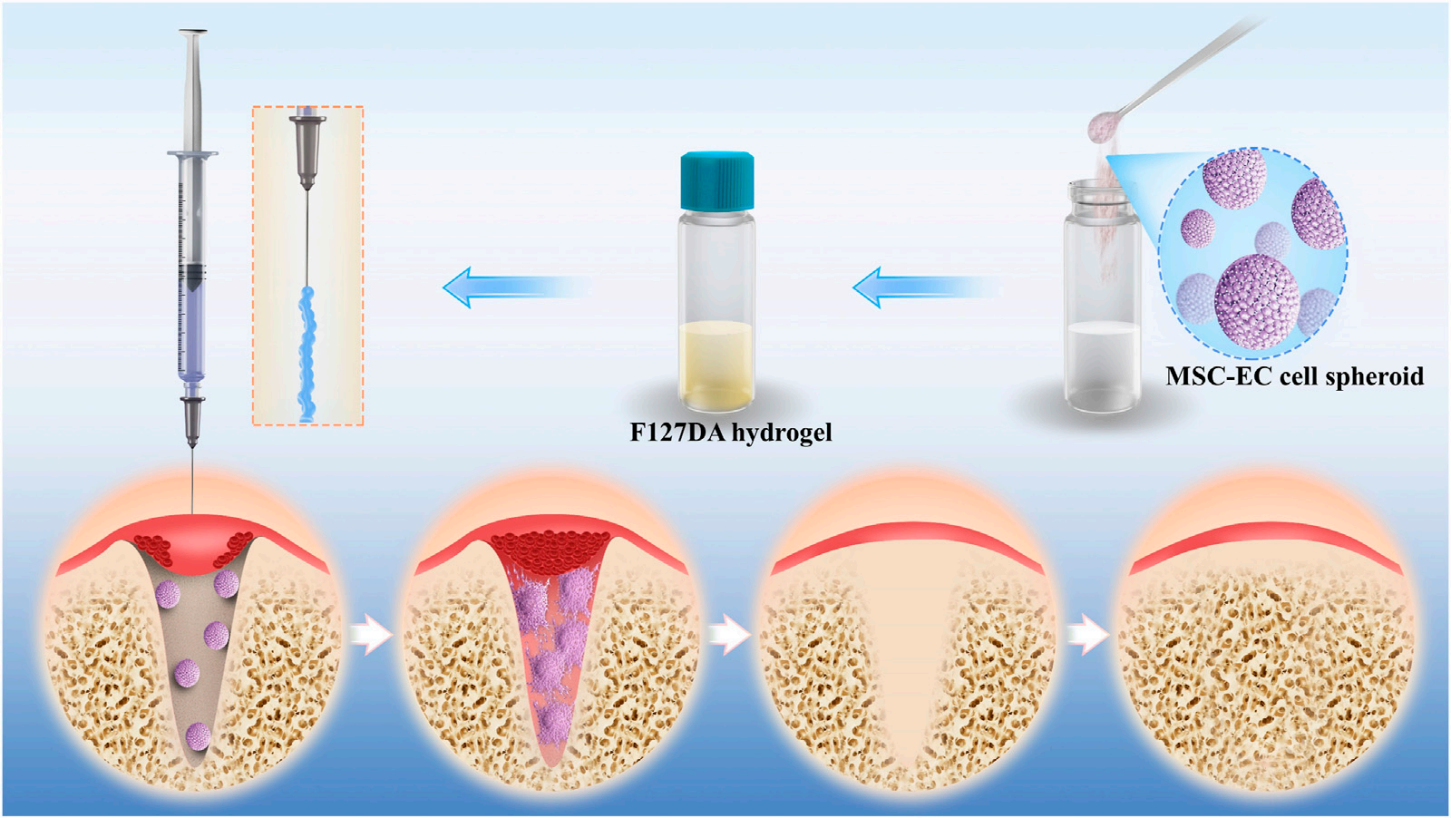
Schematic illustration of F127DA hydrogel loading with MSC-EC spheroids promoting tooth extraction socket healing.

Figure 1 illustrates the principle and procedure of the study. MSC-EC mixed spheroids were added to the uncured hydrogel to form a flowable, injectable hydrogel suspension. After injection of the suspension into the alveolar sockets, the hydrogel became more viscous at 37°C due to the temperature-sensitive property of F127DA, so that it could not flow out of the sockets easily. The hydrogels were immediately light cured to acquire a further retention. Then, cells in the spheroids actively spread in a 3D direction gradually, enhancing osteogenesis and angiogenesis, and thus promoting socket healing.

**FIGURE 2:**
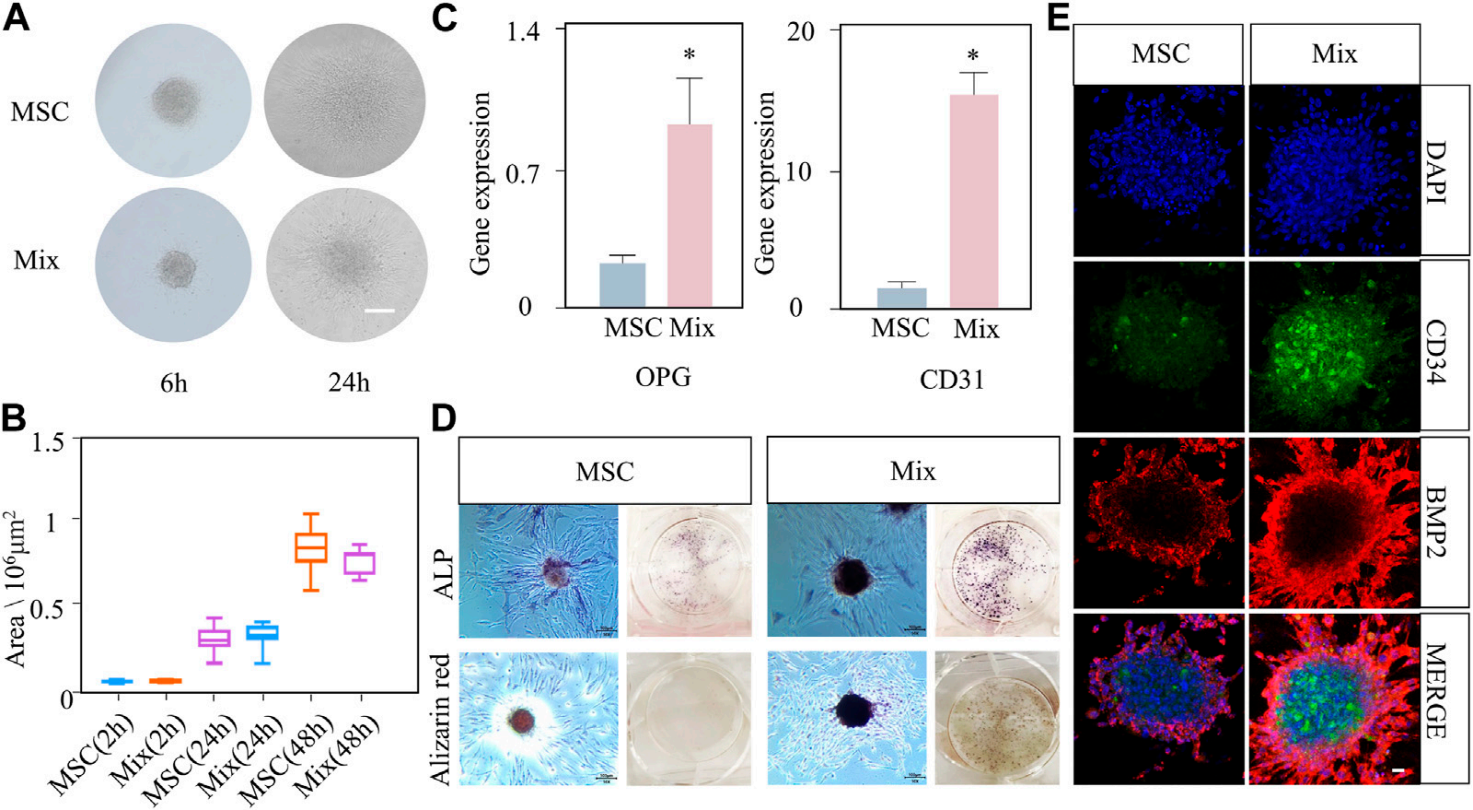
The active spreading and osteogenic abilities of MSC-EC mixed spheroids (Mix) were stronger than that of MSC spheroids. **(A, B)** Light microscope images of budding experiment, and the statistical data of spheroid area change with time in the Mix and MSC groups. Scale bar: 100 μm. **(C)** qPCR results of OPG and CD31 gene expression at 3 days of culture. **(D)** ALP staining and alizarin red staining images taken at 3 or 10 days of spheroid culture. **(E)** Immunofluorescence exhibiting the expression of CD34 and BMP2 in the Mix and MSC groups. Scale bar: 10 μm * (*p* < 0.05) indicate statistically significant differences.

Our experiment showed that the MSC-EC mixed spheroids had active spreading, osteogenesis and angiogenesis capacities. Moreover, the mixed spheroids had stronger osteogenic ability than the single MSC spheroids. In the budding experiment, light microscopy showed that only a small number of cells spread out of the spheroid at 6 h, but the length and number of buddings increased at 24 h in both groups (Figure 2A). Quantitative analysis showed that the spheroid spreading area of the two groups gradually increased with the passage of time, and reached twice at 48 h as much as that at 24 h (Figure 2B). No significant difference was found between two group at all time points. Immunofluorescence staining of cytoskeleton also showed an overall tendency of outward infiltration of the spheroids (Supplementary Figure S1). The above results indicated that mixed spheroids had the same spreading ability as the single MSC spheroids. Then, angiogenic and osteogenic potential of the spheroids was studied. PCR results showed that osteogenic and angiogenic gene markers OPG and CD31 were significantly more highly expressed in mixed spheroids (Figure 2C). ALP and alizarin red staining showed that more mature osteoblasts and calcium deposits were formed in the Mix group (Figure 2D). Immunofluorescence showed a stronger BMP-2 expression in the mixed spheroid with the introduction of CD34^+^ ECs. Interestingly, we also found that BMP-2 positive osteoblastic cells were mainly distributed at the periphery of the spheroids, while CD34^+^ endothelial cells were mainly located at the center. Together, they exhibited a special cooperative spatial distribution pattern (Figure 2E).

**FIGURE 3:**
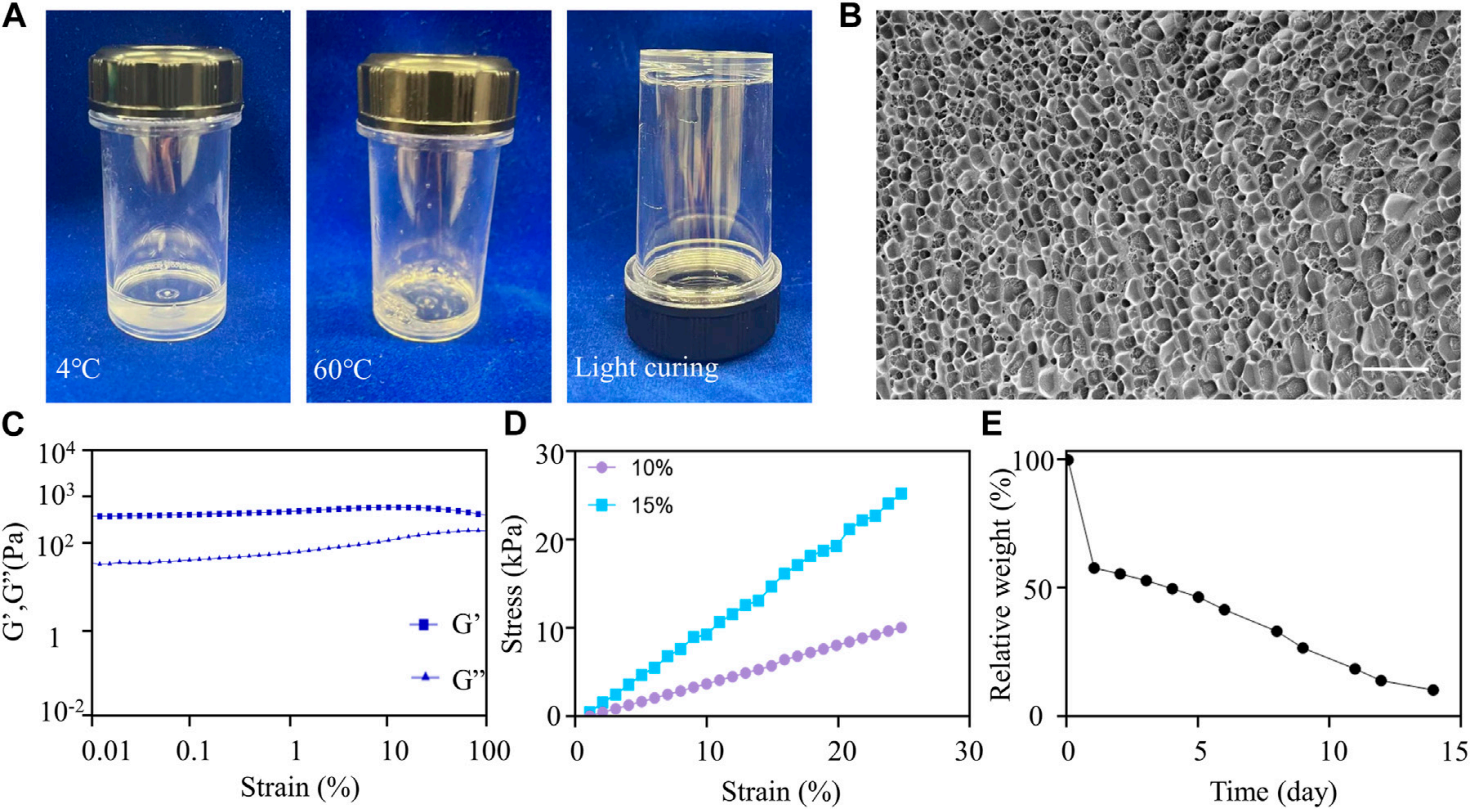
Material characterization of F127DA. **(A)** F127DA became viscous as the temperature increased and could be light cured. **(B)** Cryo-scanning electron microscope image showing the inside structure of the hydrogel. Scale bar: 10 μm. **(C)** Rheological tests showing that the hydrogel exhibited elastic solid behavior. **(D)** The compressive strength test showing the relationship between stress and strain of 10% and 15% F127DA hydrogel. **(E)** The variation curve of relative weight of the hydrogel with time at 37°C.

Based on its mechanical properties, F127DA hydrogel is an ideal scaffold material because it can support the socket, prevent bone collapse, and allow the spheroids to grow and spread. In order to test the fluidity of F127DA visually, the vial containing hydrogel was inverted upside down for seconds and was then put upright quickly. It became more difficult for F127DA solution to flow down at 60°C rather than at 4°C (Figure 3A). After light curing, F127DA hydrogel turned into a semi-solid state and could not flow down after being inverted (Figure 3A). When injecting F127DA into water at 25°C or 60°C respectively, the hydrogel became more colloidal at higher temperature (Supplementary Figure S2A). The above results indicated that F127DA hydrogel is thermosensitive, light curable and injectable. Cryo-scanning electron microscopy showed that F27DA hydrogel had a loose and porous structure. The average size of inside pores was about 4 μm (Figure 3B). The rheological test demonstrated elastic solid behavior (Figure 3C). Moreover, F127DA exhibited a strong compressive strength. The relationship between stress and strain was close to linear, so the material underwent elastic deformation with complete recovery (Figure 3D). Supplementary Figure S2B also showed good bendable and tensile properties of light cured F127DA hydrogel. The degradation experiment showed that F127DA slowly degraded at 37°C, with 50% weight of hydrogel degraded on the fourth day, and nearly 100% at the 14th day (Figure 3E). Hence, F127DA has enough time to support cell spreading, and avoids hindering bone repair due to material occupancy as well. Supplementary Figures S2C, SD showed the 3D state of spheroids in the hydrogel under light microscopy and scanning electron microscopy. It can be seen that the spheroid grew in a spherical shape and was embedded with surrounding hydrogel fibers.

**FIGURE 4:**
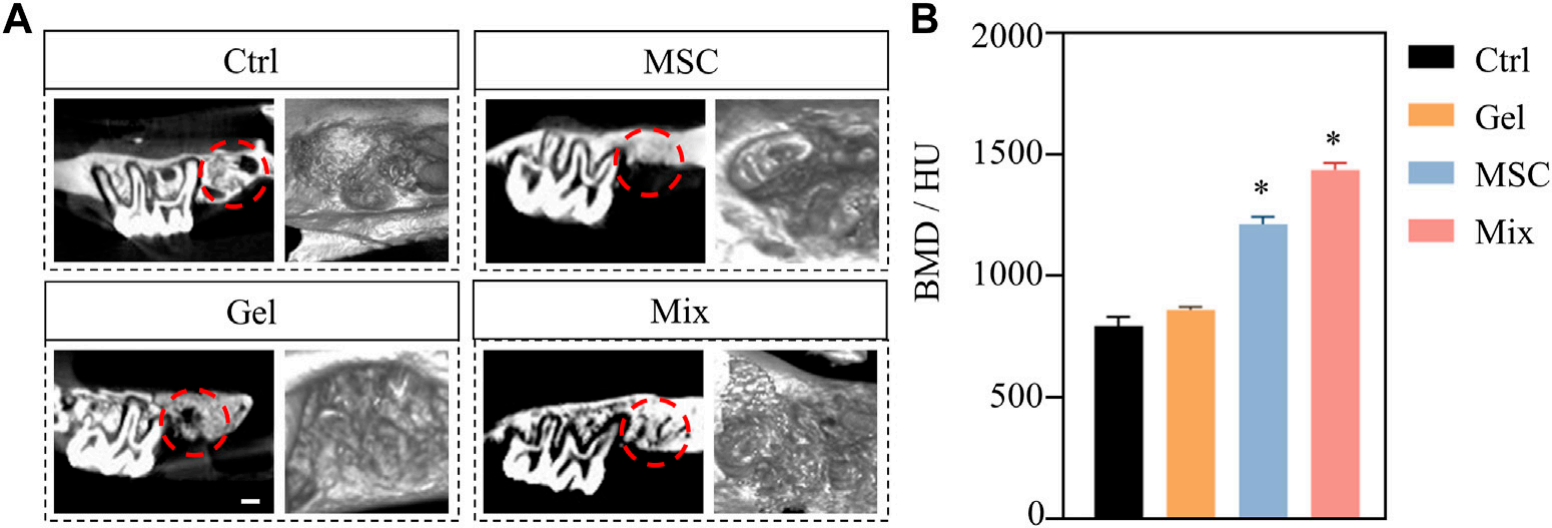
**(A)** Micro CT images of rat maxillae of Ctrl, MSC, Gel and Mix groups at 3 weeks after implantation. Scale bar: 500 μm. **(B)** The statistical results of bone mineral density (BMD) values. * (*p* < 0.05) indicate statistically significant differences.

**FIGURE 5:**
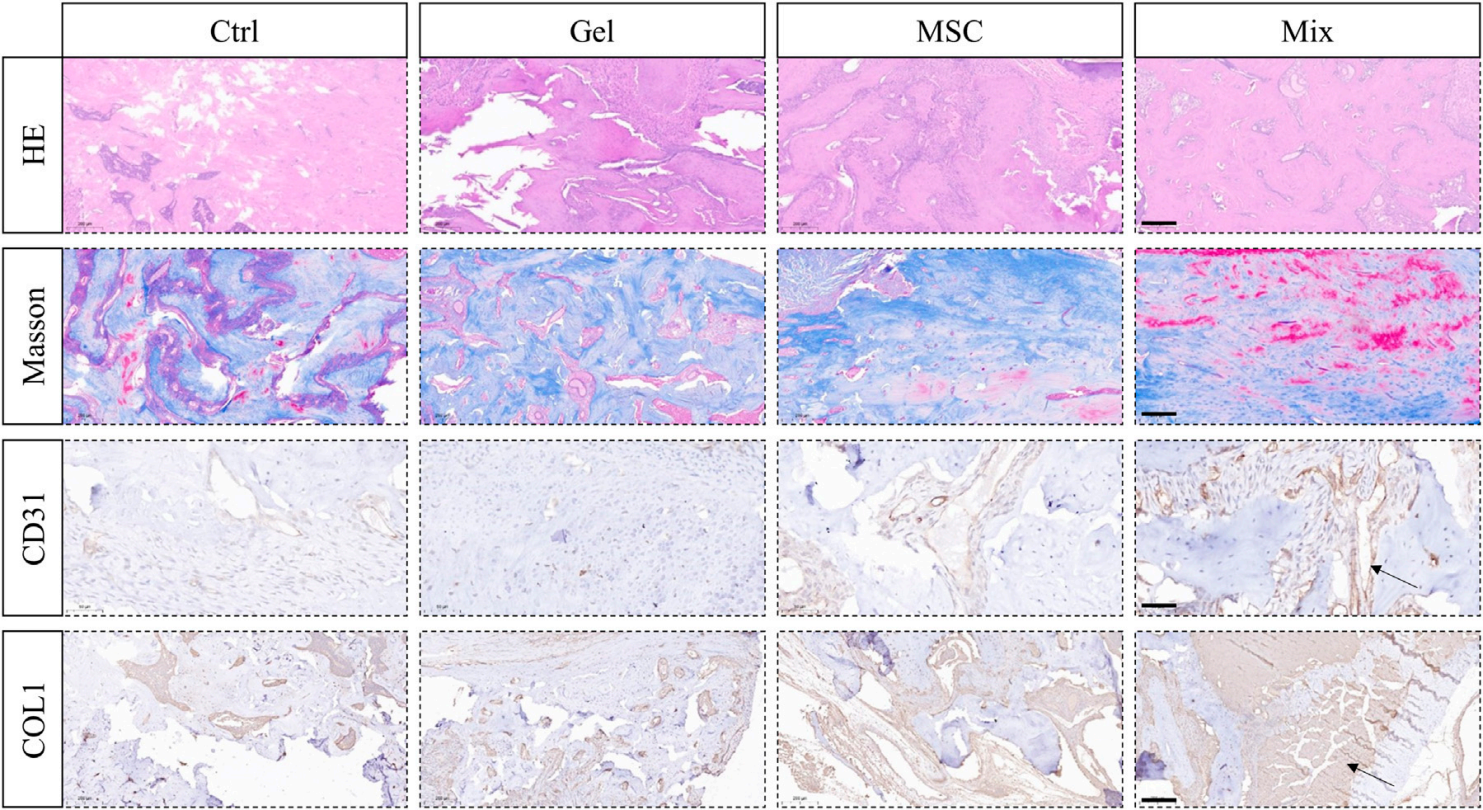
HE and Masson staining showing the trabeculae structure and collagen forming in the tooth sockets; Immunohistochemical staining of angiogenic marker CD31 and osteogenic marker COL1 in order to observe angiogenesis and osteogenesis effects. Scale bar: 200 μm (HE, Masson, COL1); 50 μm (CD31). The arrow sign: positive expression tissue.

After being loaded with MSC-EC mixed spheroids, the hydrogel was immediately implanted into the alveolar fossa of rats. We found that this MSC-EC-F127DA actively-spreading material can facilitate vascular regeneration and bone repair *in vivo*. In both Ctrl and Gel groups, Micro CT images showed poor regeneration effects and low bone density in the fossa area, and obvious bone depression could be seen by 3D reconstruction; On the other hand, the sockets of MSC group recovered well, but the level of the alveolar ridge was quite low; The restoration effect was the best in the Mix group, with the maximum bone mass and the highest alveolar ridge level at the same time. Bone mineral density (BMD) measurement also showed that BMD of the Mix group was the highest. In a word, the restoration and healing effects of MSC-EC mixed spheroids implantation was the best (Figure 4). HE staining showed that the trabeculae of the Ctrl and Gel group were loose, and there were many spaces in between; In the MSC group, the trabecular structure was more complete, bug still not dense enough; In the Mix group, the bone trabeculae were much more densely arranged and closely connected. Masson staining also showed more collagen fibers in the alveolar fossa of the Mix group. Immunohistochemical images demonstrated a higher expression of angiogenic and osteogenic markers CD31 and COL1 in the Mix group (Figure 5). All these data indicate that MSC-EC-F127DA material enhances osteogenesis and angiogenesis, and thus promote the healing of sockets.

## 4 Discussion

We developed a new superwetting material, which consists of photocurable polyester F127 diacrylate hydrogel loaded with MSC-EC mixed cell spheroids. This new material system can effectively promote bone regeneration after being injected into the tooth socket. According to previous studies, the horizontal absorption of alveolar ridge averaged 3.8 mm in width and 1.24 mm in vertical height at 6 months after tooth extraction. Moreover, the resorption of labial and buccal cortical bone becomes more significant without tooth. However, it is crucial for implantation therapy to maintain good bone contour and quality. The lack of bone contour support will often lead to attachment loss and gingival recession. Furthermore, bone quality is also crucial for determining the osseointegration effect (Isoda et al., 2012). In this study, the micro-CT results showed that the highest area ratio of woven bone was observed in the Mix group, as compared to Ctrl, Gel and MSC groups. This type of bone tissue is composed of porous bone trabecular layers, red bone marrow tissues and blood vessels, and will gradually mature into cortical bone at a later stage (Mercado-Pagán et al., 2015). In addition, we also observed that the trabecula near the alveolar crest could undergo cortical transformation, and bridge the buccal and lingual cortical bones to form a closed tooth socket. This would provide a favorable site for subsequent implantation. Histomorphology results showed that the Mix group exhibited the highest neovascularization at 3 weeks post-implantation. The generation of new blood vessels and the establishment of vascular networks are among the most important driving forces for tissue healing (Rouwkema and Khademhosseini, 2016). Adequate blood supply can provide oxygen, minerals and nutrients to the surrounding tissues, laying a solid foundation for subsequent bone healing (Percival and Richtsmeier, 2013). In conclusion, this novel superwetting material system has a strong ability to promote osteogenesis and angiogenesis, accelerate bone healing and preserve alveolar ridge after tooth extraction.

After further evaluation of the properties of this material, we found that the hydrogel has good biocompatibility, and can undergo full degradation within half a month. Moreover, the material is temperature-sensitive and gradually becomes viscous with increase of temperature. Additionally, it is also photocurable and has high strength. Hydrogels have similar biological structural characteristics to natural extracellular matrix (ECM), such as high water content, good biodegradability, high porosity and good biocompatibility (Seliktar, 2012). Therefore, hydrogels can provide a conducive microenvironment for implanted cells to colonize, grow and differentiate. Moreover, F127DA hydrogel is also slowly biodegradable, which avoids space occupation and allows bone formation. Additionally, F127DA is flowable and injectable at room temperature, and becomes more viscous at 37°C, so that the hydrogel stays in the tooth socket more easily. By light curing for 30 s, which is quite accessible at dental clinic, F127DA becomes solidified and achieves good mechanical strength. Due to its injectable and light-curable properties, the hydrogel can fill in irregular defects and provide good support for bone and soft tissue. Injection administration is also less invasive than surgeries and avoids secondary trauma caused by traditional bone transplantation (Piantanida et al., 2019). In conclusion, F127DA hydrogel is an ideal scaffold material for alveolar ridge preservation owing to its outstanding physical properties.

F127DA hydrogel is also a good scaffold for 3D cell culture. It can be seen from SEM that the hydrogel is loose and porous, and the cell spheroid can be chimed with the micelle. This structure favors the spheroids to spread outwards along the micelle. The directed migration of cell populations is the basis for cell development, wound healing and tissue regeneration (Su et al., 2018). On one hand, the excellent diffusion ability of the cell spheroids in F127DA may be due to the hydrophobic micelles, which are uneven and cross-linked with each other at the nanoscale. This structure is similar to the mastoid of the lotus leaf, and is thus beneficial for the infiltration and spreading of the spheroid. On the other hand, after bone injury, BMSCs can migrate to the damaged site and promote structural and functional recovery (Abkowitz et al., 2003). As can be seen from the immunofluorescence images, the MSC-EC mixed cell spheroids not only help reconstruct blood vessels, but also promote MSCs migration and colonization. At the same time, ECs within the spheroid improved osteogenic function of MSCs, which further enhanced bone regeneration within the tooth socket.

Compared with two-dimensional cell culture, 3D spherical cultures of MSCs can promote the release of growth factors such as Oct4, Sox2 and Nanog, which play key roles in maintaining self-renewal and multi-directional differentiation potential of stem cells (Tsai et al., 2017). In addition, MSCs express anti-apoptotic gene Bcl-2 at high levels and pro-apoptotic gene Bax at low levels during 3D spherical culture (Cheng et al., 2013). Therefore, implantation of cell spheroids instead of 2D-cultured cells prevents problems like decreased “stemness” and weak proliferative capacity. On the other hand, when the stage of restoration initiates and replace inflammatory stage during the healing process of tooth socket, mature monocytes/macrophages will differentiate into osteoclasts. The stability of osteoclast function is crucial for starting bone remodeling (Kubatzky et al., 2018). By reducing secretion of IL-1β and TNF-a, MSC spheroids decrease pro-inflammatory M1 phenotype polarization of macrophages, thereby reducing immune rejection and physiological bone resorption (Zhang et al., 2020). However, implanting MSC spheroids along cannot fully activate the osteogenic potential of MSCs and achieve satisfactory osteogenesis effect. Conventional *in vitro* osteogenic induction drugs, such as β-sodium glycerophosphate and dexamethasone, exert certain cytotoxic effects *in vivo*. Therefore, it is crucial to seek a bio-safe induction method. Some studies have shown that the joint transplantation of HUVECs and BMSCs into mice bone defects can accelerate bone healing and promote angiogenesis (Böhrnsen et al., 2021). Therefore, we hypothesized implanting MSC-EC mixed spheroids can potentially enhance osteogenesis in tooth sockets. The *in vivo* results showed that the osteogenic and angiogenic gene markers in the Mix group were significantly upregulated. The interaction between ECs and MSCs in the spheroids provides a cell-guided co-migration mechanism, and a specific pro-osteogenic and pro-angiogenic microenvironment for bone repair.

In summary, we have successfully developed a superwetting material system based on F127DA hydrogel and MSC-EC spheroids. The spheroids in this system have strong 3D active spreading capacity, and can effectively promote bone and blood vessel remodeling. The injectability, mechanical durability, and strength of the F127DA make it readily amenable and convenient for clinical applications. The MSC-EC-F127DA system, as a pro-healing material for tooth sockets, has demonstrated its potential as a supramolecular biomaterial, and can be applied to various biomedical engineering applications in the future.

## Data Availability

The original contributions presented in the study are included in the article/Supplementary Material, further inquiries can be directed to the corresponding author.
