# Supplementary figures and images for: A three-dimensional actively spreading bone repair material based on cell spheroids can facilitate the preservation of tooth extraction sockets
Source: Front Bioeng Biotechnol. 2023 Feb 27;11:1161192. doi: 10.3389/fbioe.2023.1161192 (PMC10009228; doi:10.3389/fbioe.2023.1161192)

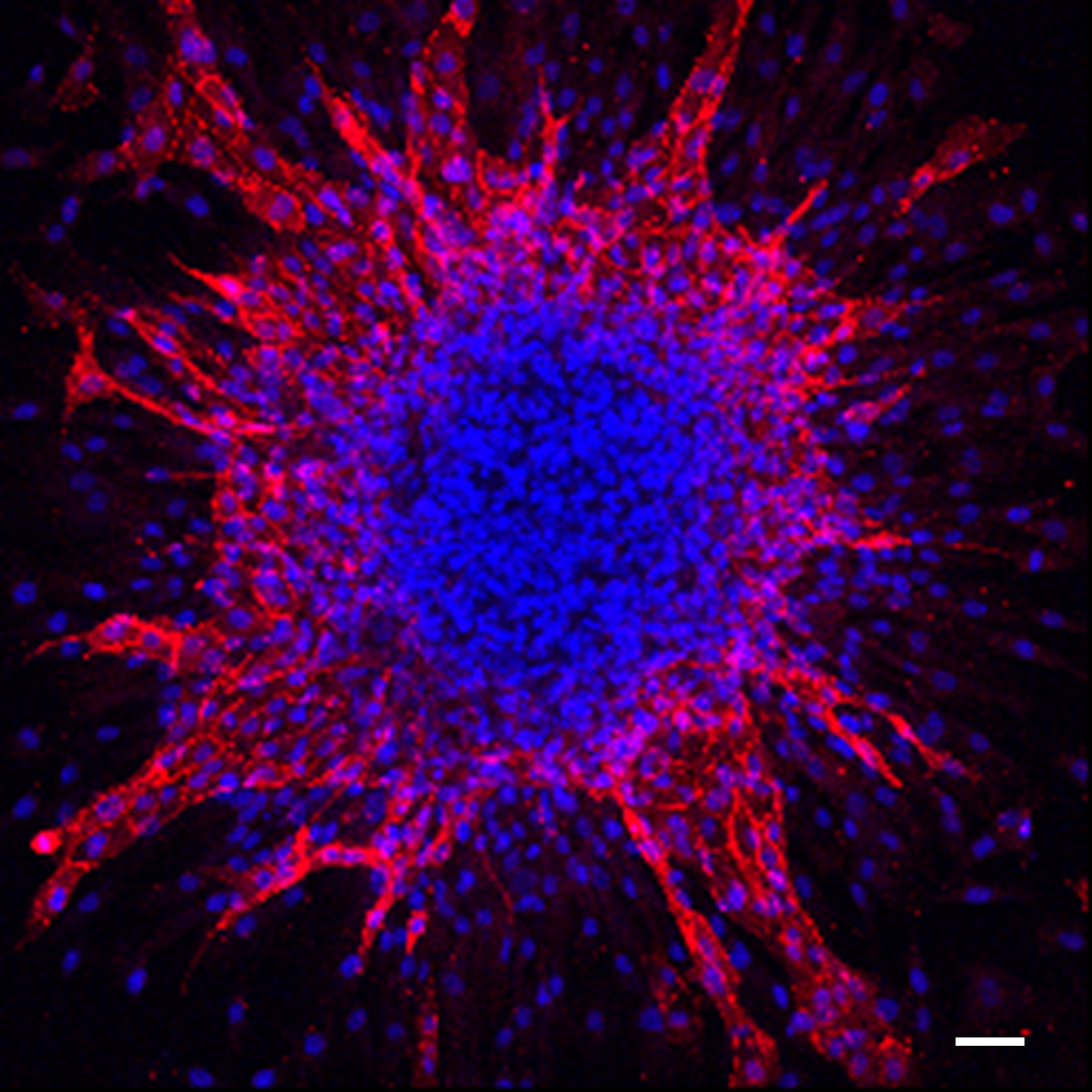

Supplement: Supplementary file 1 [file Image2.TIF]

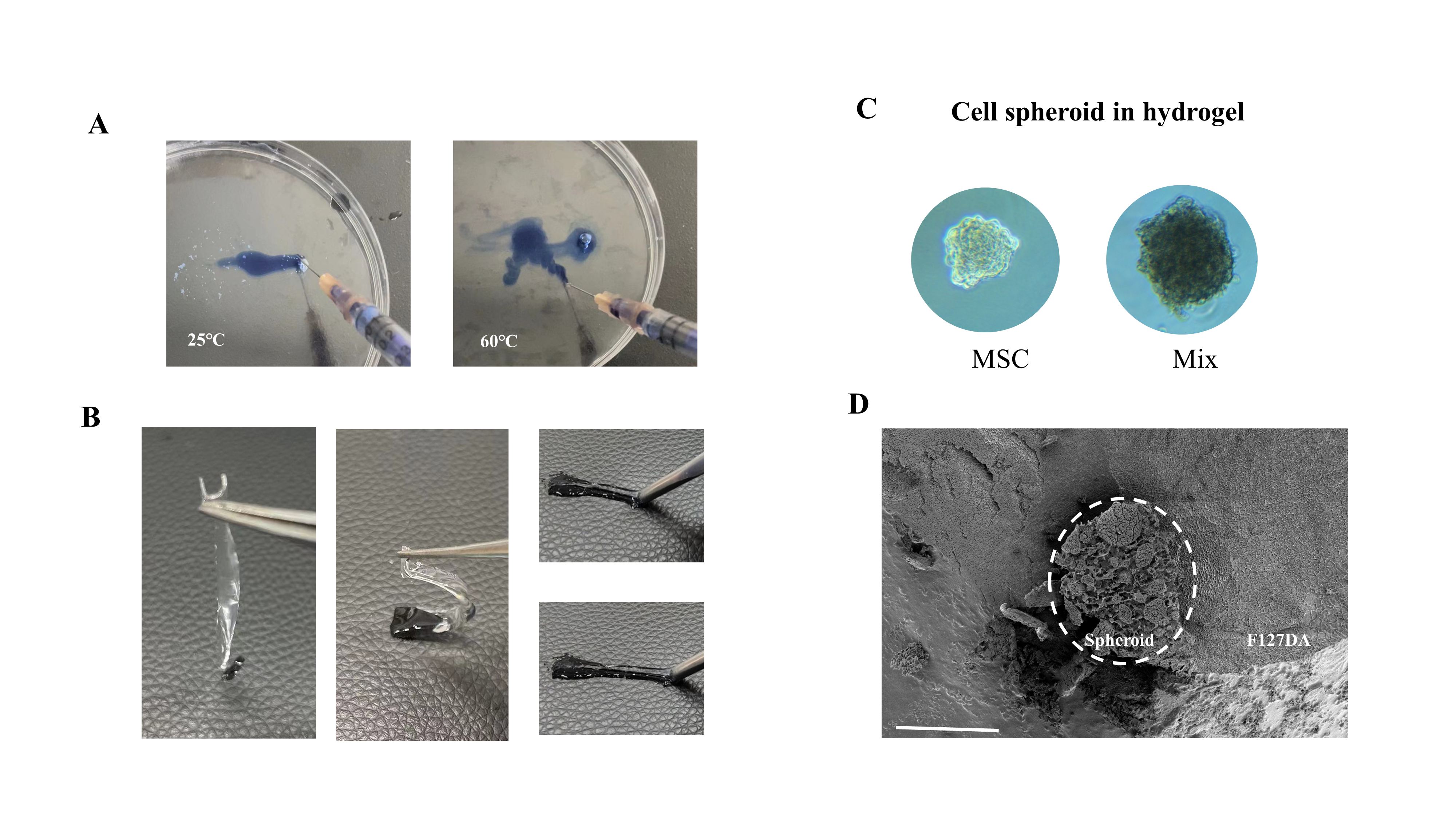

Supplement: Supplementary file 2 [file Image1.TIF]
